# Using Integrated Bioinformatics Analysis to Identify Saponin Formosanin C as a Ferroptosis Inducer in Colorectal Cancer with p53 and Oncogenic KRAS

**DOI:** 10.3390/antiox14081027

**Published:** 2025-08-21

**Authors:** Hsin-Chih Chen, Ching-Ying Chen, Pao-Yuan Wang, Pin-Yu Su, Shu-Ping Tsai, Chi-Pei Hsu, Hsiao-Sheng Liu, Chi-Ying F. Huang, Wen-Hsing Cheng, Ming-Fen Lee, Chun-Li Su

**Affiliations:** 1 Department of Human Development and Family Studies, National Taiwan Normal University, Taipei 10610, Taiwan; cxz@mail.ndmctsgh.edu.tw; 2 Instrument Center, National Defense Medical University, Taipei 11490, Taiwan; 3 Graduate Program of Nutrition Science, School of Life Science, National Taiwan Normal University, Taipei 11677, Taiwan; 61151008s@ntnu.edu.tw (C.-Y.C.); paoyuanwang@ntuh.gov.tw (P.-Y.W.); 61251016s@ntnu.edu.tw (P.-Y.S.); 61251001s@ntnu.edu.tw (S.-P.T.); 61251002s@ntnu.edu.tw (C.-P.H.); 4 Department of Dietetics, National Taiwan University Hospital, Taipei 10002, Taiwan; 5 M.Sc. Program in Tropical Medicine, College of Medicine, Kaohsiung Medical University, Kaohsiung 80708, Taiwan; hsliu713@kmu.edu.tw; 6 Center for Cancer Research, College of Medicine, Kaohsiung Medical University, Kaohsiung 80708, Taiwan; 7 Department of Microbiology and Immunology, College of Medicine, National Cheng Kung University, Tainan 70101, Taiwan; 8 Institute of Biopharmaceutical Sciences, National Yang Ming Chiao Tung University, Taipei 11211, Taiwan; cyhuang5@nycu.edu.tw; 9 Taiwan International Graduate Program in Molecular Medicine, National Yang Ming Chiao Tung University and Academia Sinica, Taipei 11211, Taiwan; 10 Department of Biotechnology and Laboratory Science in Medicine, National Yang Ming Chiao Tung University, Taipei 11211, Taiwan; 11 Department of Biochemistry, College of Medicine, Kaohsiung Medical University, Kaohsiung 80708, Taiwan; 12 Department of Nutrition and Food Sciences, Texas Woman’s University, Denton, TX 76204, USA; wcheng1@twu.edu; 13 Department of Nutrition, China Medical University, Taichung 40678, Taiwan

**Keywords:** diosgenin saponin formosanin C, gene analysis, ferroptosis, p53, KRAS, colorectal cancer

## Abstract

Ferroptosis, a form of cell death, is characterized by lipid peroxidation and is dependent on iron and reactive oxygen species (ROS). Here, through bioinformatics analysis, formosanin C was predicted to be a ferroptosis inducer in colorectal cancer (CRC) by suppressing antioxidation capacity. Indeed, formosanin C induced iron accumulation, lipid ROS formation, and ferroptosis in CRC. We found that *TP53* and *KRAS* were the second and third most frequently mutated genes in CRC and were associated with a poor prognosis. Analyses of differentially expressed genes indicated that fatty acid and labile iron levels tended to be higher in CRC than in normal tissues, suggesting the predisposition of CRC cells to ferroptosis. Transcriptomic analyses in CRC patients further identified that wild-type *TP53* and mutant *KRAS* separately favored ferroptosis. Likewise, p53 knockdown rendered HCT 116 cells less sensitive to ferroptosis, and KRAS HT-29 cells were more sensitive to ferroptosis compared with their parental counterparts. Moreover, formosanin C synergistically enhanced chemosensitivity to cisplatin, and this process was mediated by lipid ROS. Overall, our novel gene-expression screening platform allows for the efficient identification of the biological function of novel phytochemicals, and the data suggest that formosanin C is an effective ferroptosis inducer in CRC cells with p53 or oncogenic KRAS.

## 1. Introduction

Iron is an essential micronutrient; however, excessive iron is toxic, partially due to the production of ROS via the Fenton reaction [1]. Ferroptosis, an intracellular iron- and lipid ROS-dependent form of cell death proposed by Stockwell and colleagues [2], is independent of other divalent transition metal ions (Cu^2+^, Mn^2+^, Ni^2+^, and Co^2+^) and is induced by glutathione depletion, and morphologically, biochemically, and genetically different from apoptosis, necrosis, and autophagy. Consistent with these features, lipophilic antioxidants and iron chelators, but not caspase inhibitors, effectively suppress ferroptosis [2]. Interestingly, refractory cancer cells that resist targeted therapy have been reported to be vulnerable to ferroptosis [3]. Recently, ferroptosis was reported to spread across human cells over long distances at constant speeds through waves of ROS to cause tissue-scale cell death, suggesting that ferroptosis has great applicability in human pathologies, including the elimination of tumor tissues [4].

CRC is the third most common cause of cancer-related death [5]. The location of cancer affects clinical outcomes and drug responsiveness. Tumors in the proximal large intestine exhibit an advanced tumor-node-metastasis stage, microsatellite instability, with frequent mutations in *KRAS* and *BRAF*, and have a worse prognosis, whereas those in the distal large intestine show chromosomal instability and have a better prognosis [6]. Other clinical factors (i.e., lack of receiving a vascular endothelial growth factor inhibitor, exposure to fewer lines of chemotherapy, increased white blood cell count, and having the primary tumor not resected) also explain the poor prognosis [7]. Histological results determine the pathological staging of and subsequent therapies against CRC [8]. Although predictive biomarkers guide an array of treatment options early on during the tumorigenesis, nearly half of CRC patients are diagnosed at an advanced stage [9]. Although the prognosis of metastatic CRC has improved recently, resistance to chemotherapy remains an unresolved issue.

The altered expression of genes related to lipid metabolism and redox regulation in epithelial–mesenchymal transition predisposes metastatic and advanced tumors to ferroptosis during tumor progression, which promotes drug resistance [10]. Moreover, resistance to ferroptosis is a hallmark of CRC cells and leads to drug resistance, partially attributed to the absorption of iron from the intestinal lumen and allocation for growth, but not for the iron-dependent cell death pathway [11]. Therefore, potential phytochemicals that induce ferroptosis might expand the therapeutic armamentarium in oncology to treat advanced CRC. In particular, integrated bioinformatic approaches allow for the efficient identification of potential phytochemicals.

## 2. Materials and Methods

### 2.1. Genomic Data Commons—The Cancer Genome Atlas (GDC-TCGA)

The National Cancer Institute’s GDC-TCGA data portal (https://portal.gdc.cancer.gov/, accessed on 18 September 2022) is a genomics data platform characterizing over 20,000 primary cancers and the matched normal samples of 33 cancer types. Patients’ data from “The Cancer Genome Atlas Colon Adenocarcinoma (TCGA-COAD)” stored in the GDC-TCGA were selected for the analysis of most frequently mutated genes. The accession numbers of genes referenced in this study are listed in Table S1.

### 2.2. Kaplan–Meier Plotter

The relapse-free survival of colon adenocarcinoma was analyzed via the Kaplan–Meier Plotter (https://kmplot.com/analysis/, accessed on 28 September 2022) [12], a web-based tool that can be used to conduct univariate and multivariate Cox proportional hazards survival analysis. Using the platform, the patients’ DNA data on pan-cancer generated by genomic studies were selected to produce Kaplan–Meier survival plots. Additionally, the mRNA data of colon cancer patients divided into *KRAS* and *TP53* (encoding for tumor protein p53) wild-type and mutation were used to determine the expressions of ferroptosis-related genes on relapse-free survival probability.

### 2.3. University of California at Santa Cruz (UCSC) Xena Platform

The Xena platform (https://xena.ucsc.edu/, accessed on 27 September 2022) [13], which was developed by UCSC, provides functional genomic data sets for correlation analysis between genomic and/or phenotypic variables. The mRNA expression data of solid normal tissue near the tumor and cancer tissue of “GDC-TCGA Colon Cancer (COAD)” were selected to determine the differences.

### 2.4. Connectivity Map and Library of Integrated Network-Based Cellular Signatures Unified Environment (CLUE) and ConsensusPathDB (CPDB)

The CLUE platform (https://clue.io/, accessed on 17 September 2022) was used for genetic perturbational dataset analyses. CPDB (http://cpdb.molgen.mpg.de/, accessed on 17 September 2022) [14] combines 31 public resources and is used for highly promising pathway analyses [14,15]. The differentially expressed genes (DEGs; *p* < 0.05) obtained via high-throughput RNA sequencing of breast cancer MDA-MB-231 cells treated with formosanin C, as described in our previous report [16], were incorporated into CLUE for the prediction of similar mechanisms of formosanin C in CRC. All the knockdown (KD) and overexpression (OE) genes in “HT-29” (human CRC cell line) and in “summary” (cell lines of various cancer types) were applied separately to CPDB for over-represented pathway analysis.

### 2.5. Gene Set Enrichment Analysis (GSEA)

The DEGs between formosanin C-treated and vehicle-treated breast cancer MDA-MB-231 cells published in our previous report [16] were analyzed using the GSEA v.4.1.0 software [17] to determine statistically concordant significance between two biological states. The “IBRAHIM_NRF2_DOWN” gene set deposited in chemical and genetic perturbations (CGP) [18] was downloaded from the Molecular Signature Database v.7.4 (https://www.gsea-msigdb.org/gsea/msigdb/, accessed on 30 September 2022). “Ratio_of_class means” was adopted for the ranking of genes in the list.

### 2.6. The cBio Cancer Genomics Portal (Cbioportal)

The cbioportal (https://www.cbioportal.org/, accessed on 6 October 2023) is an open access dataset resource for the interactive exploration of multidimensional cancer genomics from more than 5000 tumor samples in 20 cancer studies [19]. In this platform, the mRNA expression data of colorectal adenocarcinoma cancer patients (TCGA, Pan Cancer Atlas) with wild-type or mutations of *KRAS* and *TP53* were analyzed.

### 2.7. Dependency Map (DepMap)

The DepMap (https://depmap.org/portal/, accessed on 25 December 2023) is an open access platform for the discovery of genetic information and mRNA expression levels [20,21]. Based on the hotspot and damage mutation, colorectal adenocarcinoma cell lines were divided into wild-type and mutation groups of *KRAS* and *TP53* via DepMap. Two-class comparison computed a moderated estimate of gene expression data between wild-type and mutation groups.

### 2.8. Reagents and Cell Culture

Parental human colorectal carcinoma HCT 116 (*TP53* wild-type) and HT-29 (*KRAS* wild-type) cell lines were obtained from the American Type Culture Collection (ATCC, Rockville, MD, USA). It has been well demonstrated that either point mutations or amplifications of the wild-type genes in the *RAS* family (*KRAS*, *HRAS*, and *NRAS*) turn the genes into active oncogenes [22]. The KRAS HT-29 cell line [23] was developed by transfection with a pOPI3 plasmid (Stratagene, La Jolla, CA, USA) harboring the *KRAS* gene and pHβLacINLSneo plasmid using Lipofectamine^TM^ 2000 (Invitrogen, Carlsbad, CA, USA). p53 KD HCT 116 cells were obtained by lentiviral transduction of short hairpin RNA of *TP53* (target sequence: 5′-CGGCGCACAGAGGAAGAGAAT-3′). All cell lines were cultured in Dulbecco’s modified Eagle medium (GIBCO BRL, Gaithersburg, MD, USA) supplemented with 10% fetal bovine serum (GIBCO BRL) in a humidified atmosphere with 5% CO_2_ at 37 °C. The following compounds were used to test the efficacy of ferroptosis induction in CRC cells: ferroptosis inducers [24] 1S,3R-Ras-selective lethal small molecule 3 (RSL3, Selleck Chemicals, Houston, TX, USA), erastin (Sigma, St. Louis, MO, USA), and sorafenib (Nexavar^®^, Bayer AG, Leverkusen, Germany); a CRC-targeted drug regorafenib (Stivarga^®^, Bayer AG); a CRC chemotherapy drug cisplatin (Sigma); and phytochemicals formosanin C [25], garcinielliptone FC [26], justicidin A [27], curcumin (Sigma), lupeal (Sigma), pterostilbene (Sigma), and resveratrol (Sigma). A ferroptosis inhibitor, ferrostatin-1 (Fer-1), and an iron source, ferric ammonium citrate, were obtained from Sigma.

### 2.9. Cell Population Growth Assay

The population growth of cells was evaluated using a modified colorimetric 3-[4,5-dimethylthiazol-2-yl]-2,5-diphenyltetrazolium bromide (MTT, Sigma) assay [23]. After treatment, the medium containing the tested compounds was removed to avoid color interference in the MTT assay. The absorbance was obtained at 590 nm in an ELISA Reader (Synergy HT, BioTek, Winooski, VT, USA).

### 2.10. Compound Interaction Analysis

The interaction of two compounds on the inhibition of cell population growth was calculated by combination index (CI) using the Chou–Talalay equation [28]. Data were analyzed using CompuSyn software (version 1.1.1) (ComboSyn Inc., Paramus, NJ, USA). Synergy, additivity, and antagonism were defined as CI < 1, CI = 1, and CI > 1, respectively.

### 2.11. Flow Cytometric Analysis

The formation of lipid ROS was determined by using flow cytometry [16,29] after staining the cells with 10 µM of C11-BODIPY (Thermo Fisher Scientific Inc., Waltham, MA, USA) in the dark at 37 °C for 30 min. The cells were sorted by a flow cytometer (LSRFortessa, Becton Dickinson, Lexington, KY, USA).

### 2.12. Intracellular Iron Measurement

Intracellular iron measurement was determined by using an automated cell imaging system after staining the cells with 1 µM of FerroOrange (Merck KGaA, Darmstadt, Germany) in the dark at 37 °C. The cells were detected with an ImageXpress PICO (Molecular Devices, San Jose, CA, USA).

### 2.13. Label-Free Live Cell Imaging and Analysis

Label-free live cells were analyzed by using holotomography microscopy. After treatment, the cells were detected on a Tomocube HT-X1 (Tomocube, Daejeon, Republic of Korea).

### 2.14. Western Blot Analysis

Whole cell lysates obtained by the use of the radioimmunoprecipitation assay (RIPA) lysis buffer were separated by sodium dodecyl sulfate–polyacrylamide gel electrophoresis (SDS-PAGE) and transferred to polyvinylidene fluoride membranes (Perkin Elmer, Santa Clara, CA, USA) [16]. The membranes, blocked with 5% skim milk in Tris buffer saline with Tween 20, were incubated with anti-glutathione peroxidase 4 (GPX4; Abcam, Cambridge Science Park, Cambridge, UK) and anti-glyceraldehyde-3-phosphate dehydrogenase (GAPDH; GeneTex, Irvine, CA, USA) primary antibodies and horse radish peroxidase-conjugated secondary antibodies (Invitrogen, Carlsbad, CA, USA). The signals were determined with the Biospectrum Imaging System (Universal Hood II, Bio-Rad Laboratories, Hercules, CA, USA) and analyzed using ImageJ 1.51j8 (National Institutes of Health, Bethesda, MD, USA).

### 2.15. Statistical Analysis

The results were expressed as means ± standard errors of the means (SEMs). The data were analyzed using Student’s *t*-test (SPSS software, version 14.0). A comparison with a *p* value of <0.05 was considered statistically significant.

## 3. Results

### 3.1. CRC Is Susceptible to Ferroptosis, and Poor Prognosis Is Linked to TP53 and KRAS Mutations

**Figure 1:**
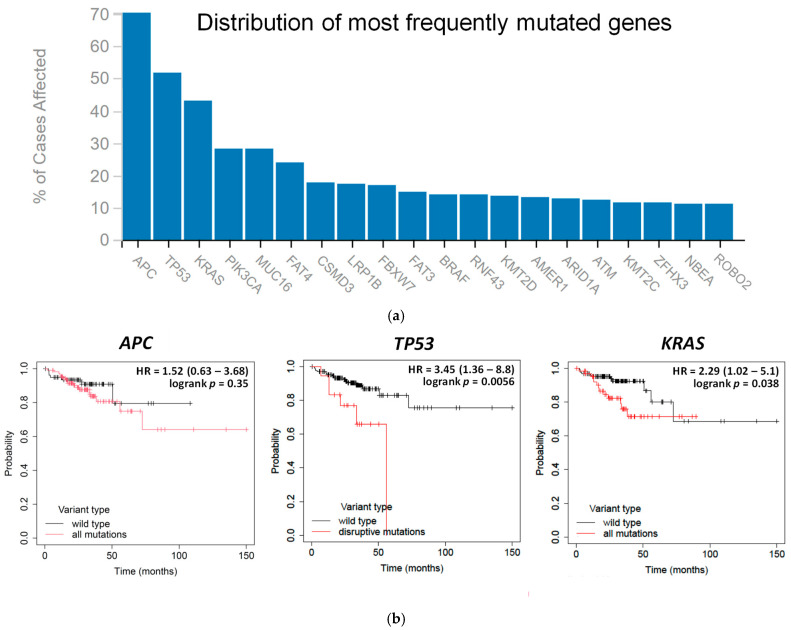

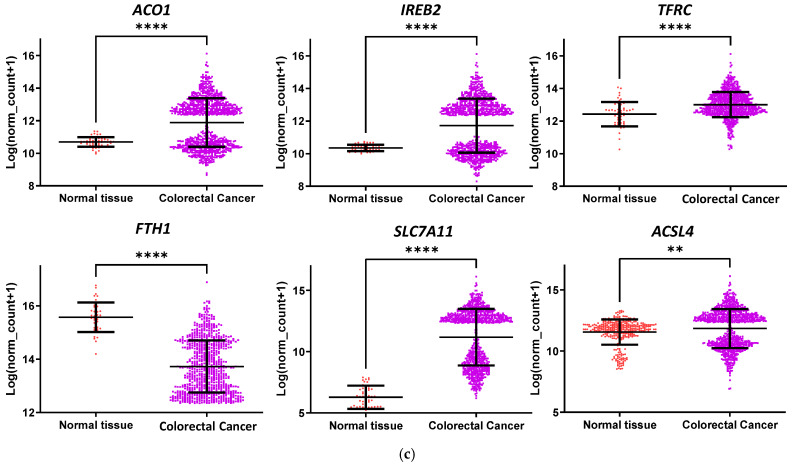
Features of human CRC using patient databases. (**a**) Mutation frequency in human CRC. Data were retrieved from GDC–TCGA (https://portal.gdc.cancer.gov/, accessed on 18 September 2022). (**b**) Prognosis of colon cancer patients. Data were analyzed by Kaplan–Meier Plotter databases (https://kmplot.com/analysis/, accessed on 28 September 2022). (**c**) mRNA expression in iron and lipid metabolism and antioxidation capacity between normal and CRC tissues. Data were analyzed via UCSC Xena (https://xena.ucsc.edu/, accessed on 27 September 2022). ** denotes *p* < 0.01. **** denotes *p* < 0.0001.

Tumors arise from normal tissues. Tumor progression is driven by a sequence of mutations that affect cell proliferation, survival, and drug resistance, which represents an important unmet clinical need. Hence, we were interested in determining the mutation rates of CRC in patients. The results obtained from the GDC-TCGA database revealed that *APC*, *TP53*, and *KRAS* were the top three most commonly mutated genes (70%, 52%, and 43%, respectively) in human CRC (Figure 1a). Because *APC* mutation, which usually occurs first in the progression of CRC and converts normal epithelium into hyperplastic epithelium, was not associated with the survival probability of patients with colon adenocarcinoma (Figure 1b), we focused on *TP53* and *KRAS* and found that their mutations were separately paralleled by a lower relapse-free survival (Figure 1b), implicating their critical roles in the progression of colorectal carcinogenesis.

There are three hallmarks of ferroptosis: oxidation of polyunsaturated fatty acid-containing phospholipids, redox-active iron, and inhibition of lipid peroxide repair [30]. In comparison with normal colorectal tissues, those from CRC patients showed an altered mRNA expression of iron-regulatory genes, including an upregulation of *ACO1* (encoding for aconitase 1 or iron response protein 1, an RNA-binding protein that upregulates intracellular iron by the inhibition of ferritin mRNA translation and degradation of transferrin receptor mRNA), *IREB2* (encoding for iron response protein 2, an RNA binding protein that upregulates intracellular iron in cooperation with iron response protein 1), and *TFRC* (encoding for transferrin receptor 1, a receptor that imports extracellular iron), and downregulation of *FTH1* (encoding for ferritin heavy chain 1, a subunit of ferritin that stores intracellular iron to limit the labile iron pool). Furthermore, CRC patients displayed higher mRNA levels of *SLC7A11* (encoding for solute carrier family 7 member 11, an anionic amino acid transport system Xc^−^ that imports cystine for glutathione biosynthesis) and *ACSL4* (encoding for acyl-CoA synthetase long chain family member 4, an arachidonic acid and CoA combination catalyzer that elevates polyunsaturated fatty acid-containing phospholipids) (Figure 1c). Altogether, these results indicate that CRC is predisposed to ferroptosis, a lipid ROS-dependent process [2], which is plausibly associated with increased fatty acid and labile iron levels. The elevated antioxidation capacity in CRC tissues by the upregulated *SLC7A11* gene suggests that lowering the effect of the antioxidative system could increase the likelihood of ferroptosis activation.

### 3.2. Saponin Formosanin C Is Predicted to Trigger Ferroptosis in CRC Cells via Diminished Antioxidation Capacity

**Figure 2:**
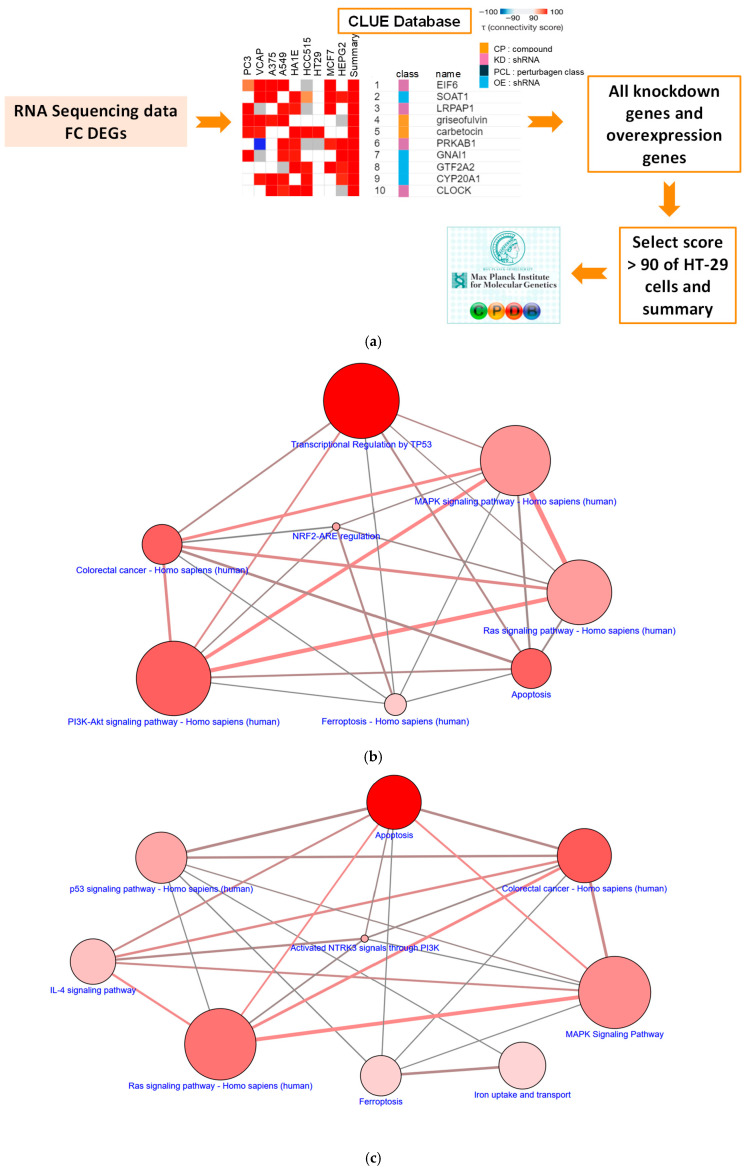

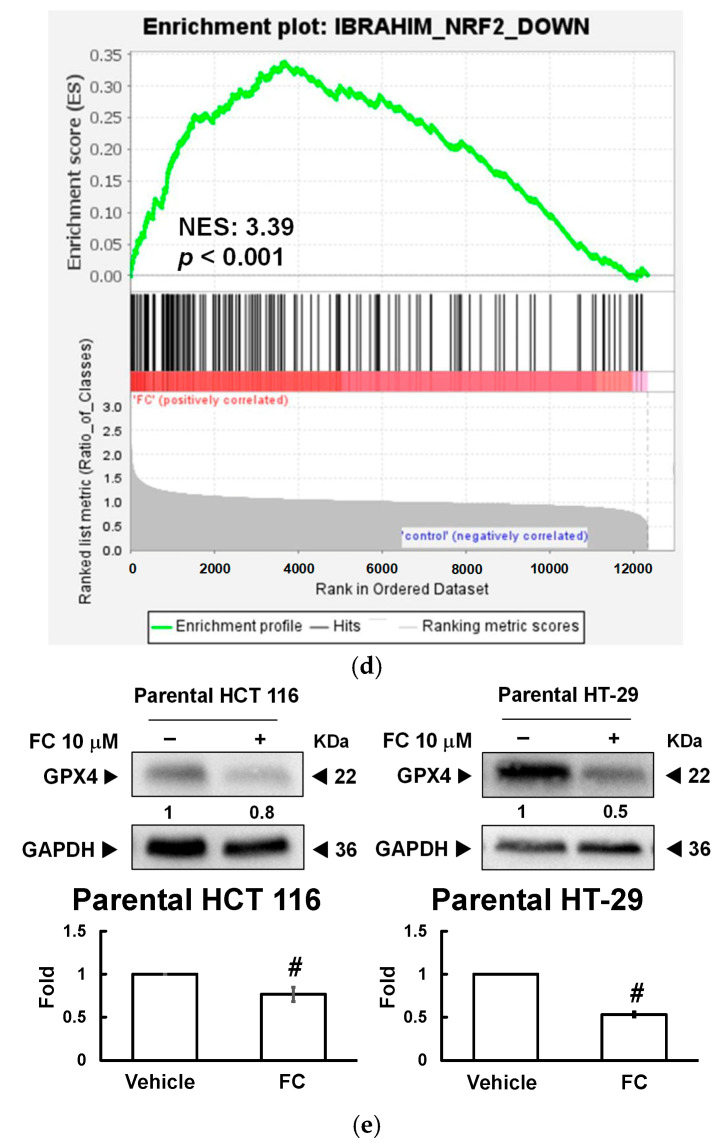
Bioinformatics analysis of the biological function of formosanin C in CRC cells. (**a**) The flow chart predicting potential mechanisms of formosanin C in human CRC via CLUE (https://clue.io/, accessed on 17 September 2022) and CPDB (http://cpdb.molgen.mpg.de/, accessed on 17 September 2022). (**b**) The over-represented pathways through analysis of the KD and OE DEGs in human CRC “HT-29” cells via CPDB. (**c**) The over-represented pathways through analysis of the KD and OE DEGs in “summary” via CPDB. The node size denotes the entity number of genes in the pathway. The color intensity of the node is positively correlated with statistical significance. The darker the color is, the stronger the statistical significance. Two nodes are connected by a line if they share genes. The line width reflects the relative overlapping genes. (**d**) Formosanin C-suppressed ROS detoxification NRF2 gene set using GSEA, accessed on 30 September 2022. (**e**) Cells received treatments of FC for 28 h. Whole cell lysates were subjected to Western blot analysis using an anti-GPX4 antibody. A GAPDH antibody was used as an internal control. The intensity of each protein expression band was quantified through densitometry normalization to that of GAPDH, with the control level arbitrarily set to 1. # Compared to the corresponding vehicle control, *p* < 0.05. FC denotes formosanin C. NES denotes normalized enrichment score.

Using transcriptomic data from one cancer type, we uncovered the novel biological function of small molecules across cancer types via our innovative gene screening platforms, as described in our previous research [31,32,33]. Through such an integrated analysis of DEGs and pathways (Figure 2a), the molecular actions of the phytochemical formosanin C in CRC (“HT29”, Figure 2b) and in the nine cancer cells altogether (“summary”, Figure 2c) were identified to be associated with “Ferroptosis–Homo sapiens (human)” and “Ferroptosis”, respectively. The result of GSEA further revealed significant enrichment and showed that formosanin C was positively (NES > 0) correlated with the “IBRAHIM_NRF2_DOWN” gene set (Figure 2d), the only significant gene set that seems to be associated with ferroptosis. Indeed, formosanin C reduced protein expression of GPX4, a transcription target of nuclear factor erythroid 2-related factor 2 (NRF2), in both HCT 116 and HT-29 cell lines (Figure 2e). NRF2 is known as a ROS detoxification protein [34]. These in silico prediction and experimental validation results suggest that formosanin C-induced ferroptosis may be plausibly ROS-mediated in CRC cells.

### 3.3. Saponin Formosanin C-Induced Ferroptosis Is Indeed Associated with Lipid ROS Formation

**Figure 3:**
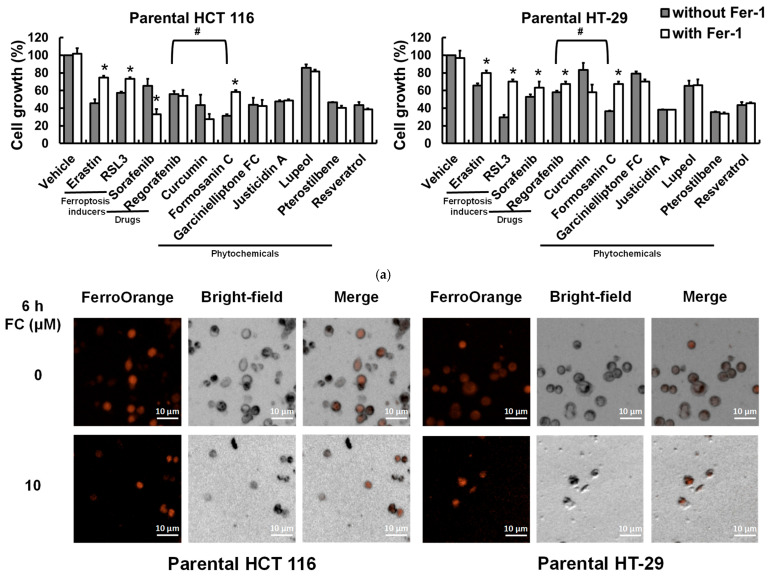

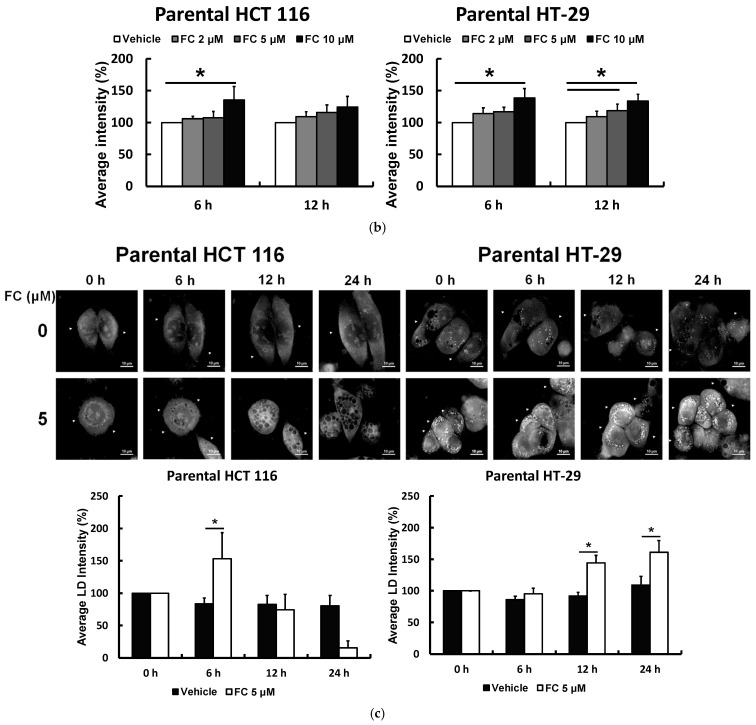

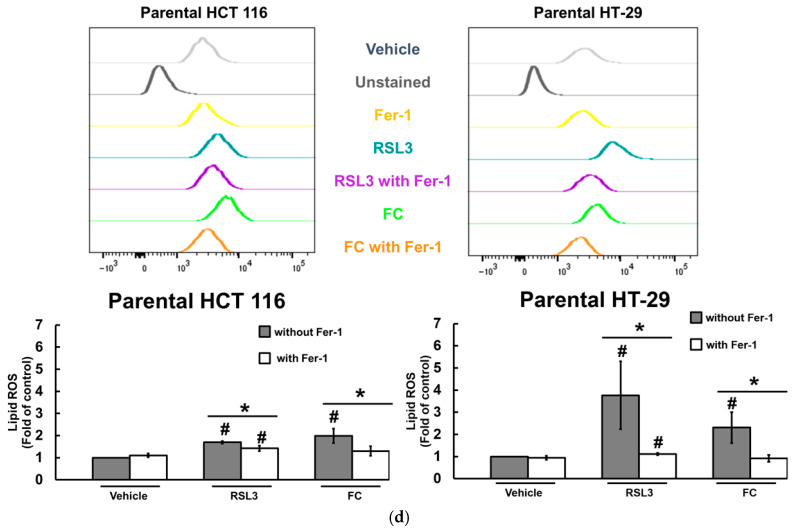
Formosanin C-induced ferroptosis in CRC cells. (**a**) Growth inhibition by anticancer drugs and seven phytochemicals. Cells were treated with erastin (10 μM), RSL3 (5 μM), sorafenib (10 μM), regorafenib (10 μM), curcumin (20 μM), formosanin C (10 μM), garcinielliptone FC (20 μM), justicidin A (10 μM), lupeol (100 μM), pterostilbene (100 μM), and resveratrol (100 μM) separately in the presence or absence of ferroptosis inhibitor ferrostatin-1 (Fer-1; 5 μM) for 48 h. Population cell growth was analyzed by MTT assay. * Compared to without Fer-1, *p* < 0.05. # denotes *p* < 0.05. (**b**) Formosanin C-induced intracellular iron accumulation. Cells were treated with formosanin C (0–10 μM) for 6 and 12 h. Intracellular iron was analyzed after staining the cells with FerroOrange using an automated cell imaging system. * denotes *p* < 0.05. (**c**) Formosanin C-elevated lipid droplets. Cells were treated with formosanin C (5 μM) for 0–24 h. Changes in the number of lipid droplets were analyzed using holotomography microscopy. The triangle indicates lipid droplets. * denotes *p* < 0.05. LD denotes lipid droplet. (**d**) Formosanin C-induced lipid ROS formation. Cells were treated with RSL3 (5 μM) and formosanin C (5 μM) separately in the presence and absence of Fer-1 (5 μM) for 48 h. Lipid ROS were determined after staining the cells with C11-BODIPY using flow cytometry. The higher the intensity (the peak shifts to the right) of C11-BODIPY fluorescence is, the richer the lipid ROS are. * denotes *p* < 0.05. # Compared to corresponding vehicle control, *p* < 0.05. FC denotes formosanin C.

Next, treatment of human CRC HCT 116 and HT-29 cells with ferroptosis inducers erastin (a system Xc^−^ inhibitor) [2] or RSL3 (a GPX4 activity inhibitor) [35] reduced cell growth, and the effects were alleviated by the ferroptosis-specific inhibitor ferrostatin-1 (Fer-1, an Fe^2+^-dependent lipid ROS scavenger [36]; Figure 3a). These observations were in line with our gene analysis results using a patient database (Figure 1c), suggesting the vulnerability of CRC to ferroptosis. Sorafenib and regorafenib are U.S. Food and Drug Administration-approved targeted therapy drugs for advanced hepatocellular carcinoma [37] and CRC [38], respectively. It is noteworthy that the reduced cell growth achieved by regorafenib was less effective than that achieved by formosanin C in both cell lines (Figure 3a), suggesting that formosanin C was more effective than the targeted therapy for these two CRC cells under the experimental conditions. Sorafenib is a ferroptosis inducer [24]. The population growth inhibitory effects of these two anticancer drugs were significantly reversed by Fer-1 in HT-29 but not HCT 116 cells. Although the seven tested phytochemicals (curcumin, formosanin C, garcinielliptone FC, justicidin A, lupeol, pterostilbene, and resveratrol) reduced cell growth to varying degrees, only formosanin C-inhibited cell growth could be significantly reversed by Fer-1 in both cell lines. Furthermore, formosanin C caused a significant labile iron accumulation in both cell lines as early as 6 h into treatment, and the elevated iron sustained to a later time point (12 h) in HT-29 cells (Figure 3b). Recently, lipid droplet accumulation has been identified as a novel hallmark of ferroptosis [39]. An increase in lipid droplets by formosanin C was observed in both cell lines (Figure 3c). These data corroborated our prediction via integrated bioinformatics analysis (Figure 2b) and demonstrated that formosanin C was a potent ferroptosis inducer in CRC cells. The observation of reduced formosanin C-induced lipid ROS levels by Fer-1 treatment in both cell lines (Figure 3d) was consistent with the notion of ROS involvement in formosanin C-induced ferroptosis in CRC cells (Figure 2d).

### 3.4. Wild-Type TP53 and Mutant KRAS Separately Favors Ferroptosis, and p53 and Oncogenic KRAS Sensitize CRC Cells to Formosanin C

**Figure 4:**
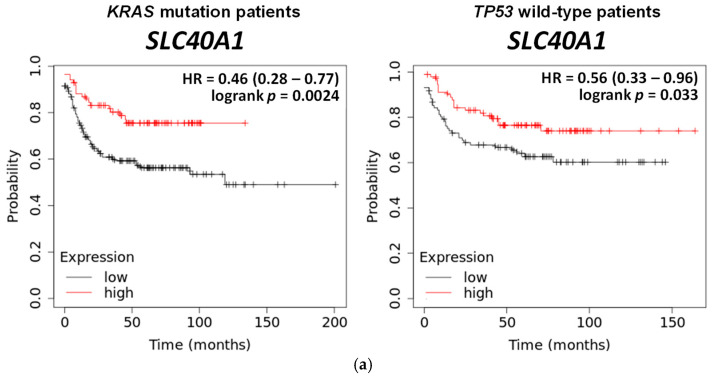

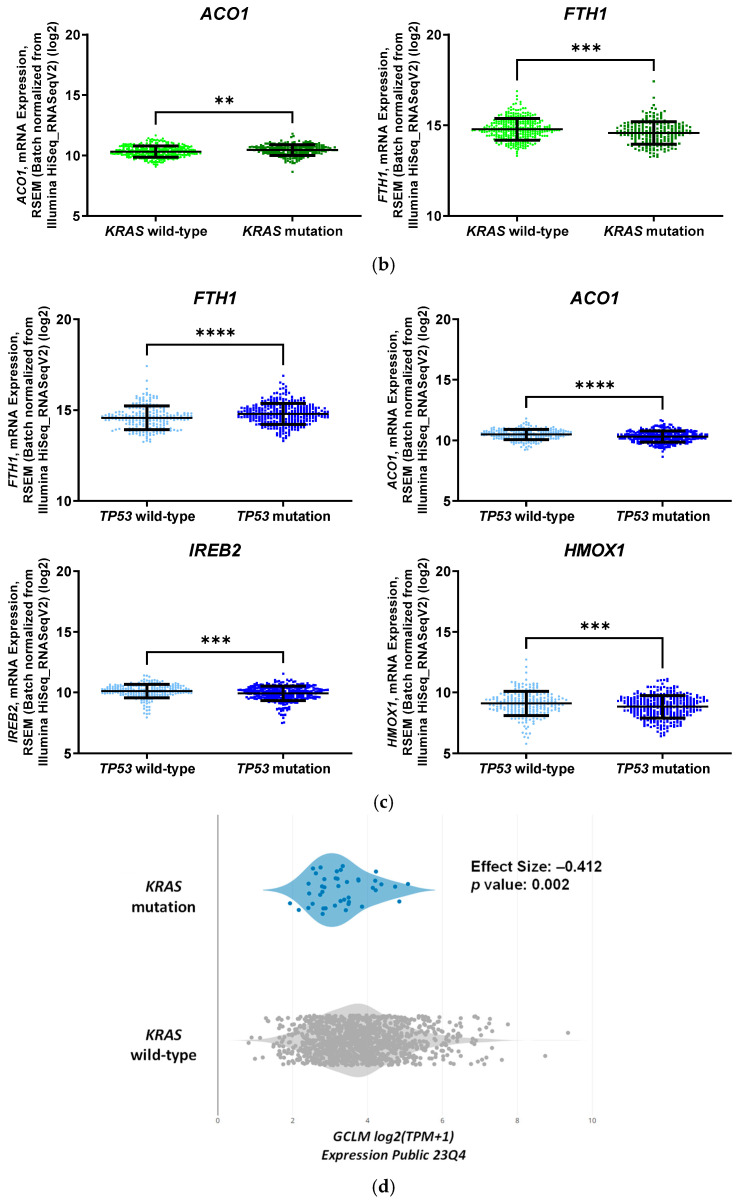

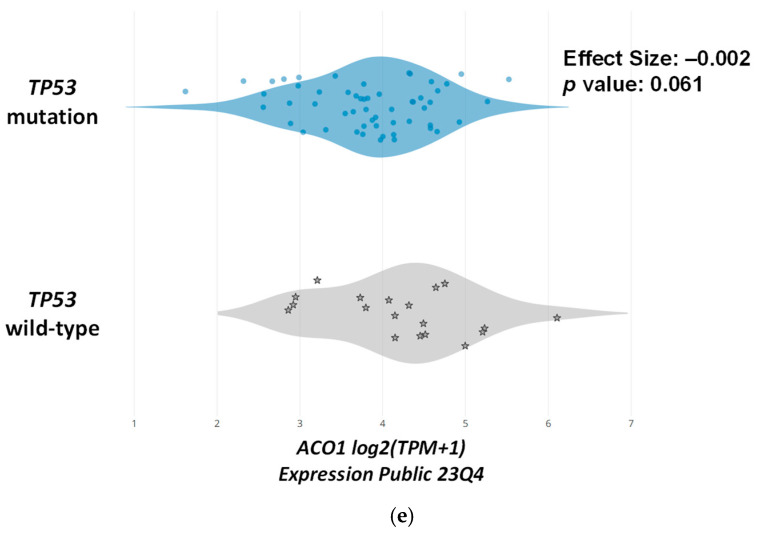
Roles of *KRAS* and *TP53* genes in ferroptosis. (**a**) Prognosis of colon cancer patients with mutant *KRAS* and wild-type *TP53*. Data were analyzed by Kaplan–Meier Plotter databases (https://kmplot.com/analysis/, accessed on 4 September 2023). Iron metabolism gene expressions in colorectal adenocarcinoma cancer tissues with wild-type vs. mutant *KRAS* (**b**) and wild-type vs. mutant *TP53* (**c**). Data were analyzed via cbioportal (https://www.cbioportal.org/, accessed on 6 October 2023). **, ***, and **** denote *p* < 0.01, 0.001, and 0.0001, respectively. (**d**) Expression of ferroptosis-related gene in colorectal adenocarcinoma cell lines with *KRAS* wild-type and mutation. (**e**) Expression of ferroptosis-related gene in colorectal adenocarcinoma cell lines with wild-type and mutant *TP53*. Data were analyzed via DepMap (https://depmap.org/portal/, accessed on 25 December 2023). *KRAS* oncogene was separated by hotspot mutation, and *TP53* tumor suppressor gene was separated by damage mutation.

**Figure 5:**
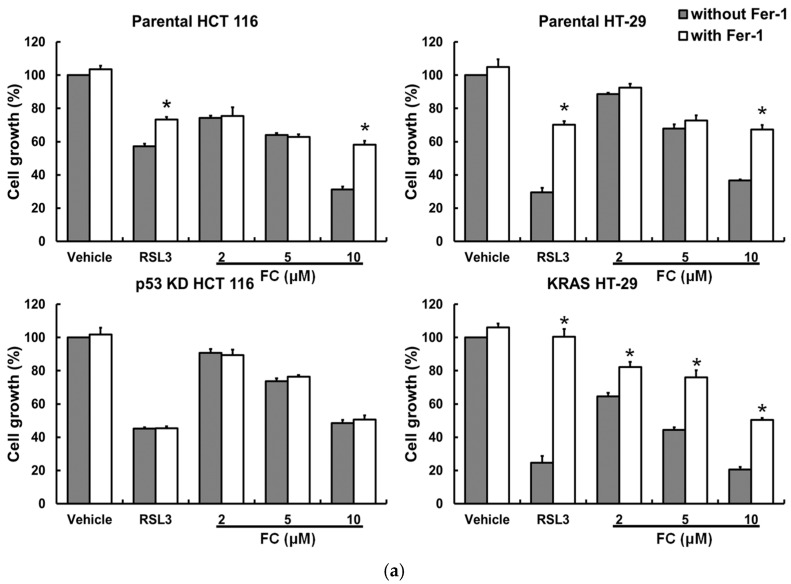

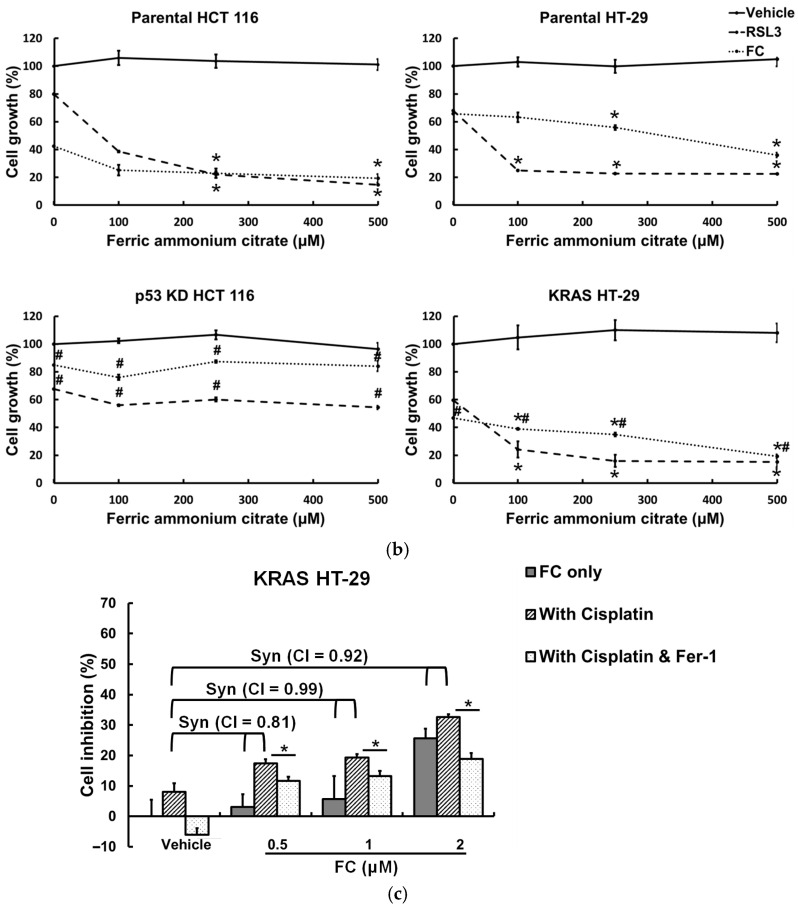
The effect of p53 and oncogenic KRAS proteins on ferroptosis. (**a**) p53 KD HCT 116 cells are less sensitive and KRAS HT-29 cells are more sensitive to ferroptosis than their corresponding parental cell lines. Cells were treated with RSL3 (5 μM) and formosanin C separately in the presence or absence of ferroptosis inhibitor ferrostatin-1 (Fer-1; 5 μM) for 48 h. * Compared to without Fer-1, *p* < 0.05. (**b**) Iron-enhanced cell growth inhibition in response to formosanin C. Cells were treated with RSL3 (1 μM) and formosanin C (2 μM) separately in the presence or absence of ferric ammonium citrate for 48 h. Population cell growth was analyzed by MTT assay. * Compared to 0 μM of ferric ammonium citrate treatment, *p* < 0.05. # Compared to the corresponding parental cells under the same experimental conditions, *p* < 0.05. FC denotes formosanin C. (**c**) Formosanin C-enhanced chemosensitivity of cisplatin. Cells were treated with the phytochemical formosanin C, anticancer drug cisplatin (5 μM), and/or ferroptosis inhibitor ferrostatin-1 (Fer-1; 5 μM) for 48 h. Population cell growth was determined by MTT assay. * denotes *p* < 0.05. The value of CI indicates antagonism when greater than 1, additivity when equal to 1, and synergism when smaller than 1. Syn denotes synergism.

In addition to suggesting critical roles of *TP53* and *KRAS* genes in the tumorigenesis of CRC (Figure 1), results from the Kaplan–Meier Plotter database further showed that a lower expression of the *SLC40A1* gene (solute carrier family 40 member 1, encoding for the protein that is involved in iron export to the outside of the cell) in colon cancer patients with wild-type *TP53* or mutant *KRAS* had a lower relapse-free survival (Figure 4a). These results suggest that such colon cancer patients with a lower survival rate tend to increasingly accumulate labile iron in the cancer tissue, and are thus predisposed to ferroptosis. Compared to colorectal adenocarcinoma cancer patients with wild-type *KRAS*, those with *KRAS* mutations expressed higher and lower mRNA levels of iron-regulatory *ACO1* and *FTH1* (Figure 4b), respectively, suggesting that elevated cellular redox-active iron saturation might occur in patients with *KRAS* mutations. In addition, colorectal adenocarcinoma cancer patients with wild-type *TP53* showed upregulated expressions of *ACO1, IREB2*, and *HMOX1* (encoding for heme oxygenase 1, the enzyme that mediates the degradation of heme) and downregulation of *FTH1* in comparison with patients with mutant *TP53* (Figure 4c), implying that patients with wild-type *TP53* may be prone to ferroptosis due to enriched cellular redox-active iron. *hmox* (encoding for the glutamate–cysteine ligase modifier subunit) is part of glutamate–cysteine ligase, the first rate-limiting enzyme of glutathione synthesis. Analyses of colorectal adenocarcinoma cell lines through the DepMap database showed that colorectal adenocarcinoma cells with *KRAS* mutants had a lower expression of *GCLM* (Figure 4d), and those with wild-type *TP53* tended to have a higher expression of *ACO1* (Figure 4e), implying that colorectal adenocarcinoma cells with *KRAS* mutations might have a lower glutathione production and thus limit antioxidative capacity, and that with wild-type *TP53* tended to increase cellular redox-active iron levels to promote ferroptosis. These results suggest that wild-type *TP53* and mutant *KRAS* may potentially increase the sensitivity of CRC cells to ferroptosis. To confirm our predictions in silico, HCT 116 (wild-type *TP53*) with p53 KD and HT-29 (wild-type *KRAS*) overexpressing KRAS were developed to test their roles in ferroptosis in CRC cells. It has been well demonstrated that either point mutations or amplifications of the wild-type genes in the *RAS* family (*KRAS*, *HRAS*, and *NRAS*) turn the genes into active oncogenes [22]. As shown in Figure 5a, p53 KD HCT 116 cells were less sensitive and KRAS HT-29 cells were more sensitive to formosanin C-induced ferroptosis than their corresponding parental counterparts. These changes were characterized by a lack of influence on cell growth in p53 KD HCT 116 cells and the attenuation of cell growth in parental HCT 116 cells at 10 μM of formosanin C in the presence of Fer-1, as well as the attenuation of cell growth by Fer-1 at a lower concentration (2 μM) of formosanin C in KRAS HT-29 than in parental HT-29 cells (10 μM). Furthermore, ferric ammonium citrate (an iron source) sensitized both parental HCT 116 and HT-29 cells to RSL3- or formosanin C-induced ferroptosis. The degree of sensitization was less pronounced in p53 KD HCT 116 cells and more effective in KRAS HT-29 cells in response to formosanin C (Figure 5b). Taken together, these results suggest that formosanin C may be an effective ferroptosis inducer in CRC, and p53 and oncogenic KRAS can separately enhance the vulnerability of CRC cells to formosanin C-induced ferroptosis.

### 3.5. Formosanin C Treatment Sensitizes KRAS HT-29 Cells to Cisplatin

Among the four tested cell lines, KRAS HT-29 cells were the most sensitive to formosanin C-induced ferroptosis (Figure 5a). The platinum-containing cisplatin has been used to treat a broad spectrum of cancers for decades; however, the nephrotoxicity of cisplatin limits its application [40]. A synergistic effect in cell growth inhibition was observed when formosanin C was used to treat KRAS HT-29 cells in combination with cisplatin, and the addition of Fer-1 attenuated the cell growth inhibition effect (Figure 5c), indicating formosanin C enhanced chemosensitivity to cisplatin by the induction of ferroptosis in KRAS HT-29 cells. These results implicate the potential effect of formosanin C in treating CRC patients with oncogenic KRAS (Figure 5), which shows poor prognosis (Figure 1b).

## 4. Discussion

The CLUE database contains transcriptional expression profiles of cells encompassing more than 5000 genetic perturbations in response to tens of thousands of compounds. This platform can be used for drug discovery and mechanism exploration by connecting differential expression gene signatures to compounds, genetic perturbations, and diseases [41,42]. The disease-associated gene signatures can be compared with the signature profiles of compounds to reveal potential drug lists, even for profiles from different cancer types. The system uses a nonparametric, rank-based algorithm to calculate a score that indicates the degree of similarity or dissimilarity between query tags (or patient signatures) and profile tags (or compound signatures). A strong positive connectivity score (similarity) indicates a similar effect, while a strong negative connectivity score (dissimilarity) shows an opposing mechanism. The target genes from the results of the CLUE analysis can be linked to CPDB, which contains interactions that have been curated from the literature, to obtain highly promising pathways. Our predictions discover formosanin C-induced ferroptosis in CRC cells and other cancer types (Figure 2), and formosanin C indeed triggers ferroptosis in cells of CRC (Figure 3 and Figure 5), hepatocellular carcinoma [29], and triple-negative breast cancer [16]. These results suggest that our innovative gene screening platforms are effective in the identification of compound-disease relationships and molecular actions of compounds in cancer cells.

Among the four sub-groups (*BRAF*-mutated; *KRAS*-mutated codons 12-13 only; any of the *KRAS* codons 61-146, *PIK3CA*, or *NRAS* mutations; and wild-type cells) of patients with metastatic CRC, those with *BRAF* (7.6 months) or *KRAS* (16.7 months) mutations had a shorter survival and the patients without those mutations had the best prognosis (27.7 months) [7]. These data suggest a negative prognostic role of *BRAF* and *KRAS* mutation separately in mutational profiling (*KRAS*, *BRAF*, *NRAS*, and *PI3KCA*) of the EGFR pathway, and the re-sensitization of *BRAF*- and/or *KRAS*-mutated CRC has prominent clinical implications. Tumors prevent ferroptosis by lowering polyunsaturated fatty acid-containing phospholipid and lipid peroxidation, raising the storage of iron from labile iron pool, and enhancing antioxidative defense systems [10]. Changes in the metabolism of amino acids, fatty acids, and iron influence ferroptosis. The activation of alternative cell death pathways provides an opportunity to overcome the drug resistance associated with existing chemotherapeutic agents. Although ferroptosis was first observed in *RAS*-mutant cancer cells [2], *RAS* mutations were also known to contribute to ferroptosis resistance in human rhabdomyosarcoma cells [43]. Our data, using isogenic cell lines, demonstrated that CRC cells with oncogenic KRAS were prone to phytochemical formosanin C-induced ferroptosis (Figure 5), and this effect further enhanced the chemosensitivity of the cells to the anticancer drug cisplatin (Figure 5c). Bromelain, a naturally occurring compound derived from pineapple stems, has been reported to induce ferroptosis in *KRAS*-mutant CRC cells via the upregulation of ACSL4 expression [44]. These observations suggest that ferroptosis could be targeted to overcome the resistance of CRC cells with oncogenic KRAS to anti-EGFR therapies [7].

The tumor suppressor p53, a transcription factor, plays a dual role in ferroptosis. The anti-ferroptosis effect of p53 can be triggered by its binding to DPP4, which prevents the interaction of the DPP4 with NOX1 to form a membrane-bound complex that transports electrons across the plasma membrane to upregulate intracellular ROS [34]. p53-mediated reduced expression of SLC7A11, a subunit of xCT, which imports cystine for glutathione formation, contributes to its pro-ferroptosis effect [34]. By using isogenic cell lines, our results demonstrate that p53 may play a pro-ferroptosis role in CRC cells in response to the ferroptosis inducer RSL3 and the phytochemical formosanin C (Figure 5).

Formosanin C, also known as Paris saponin II, is a diosgenin saponin [45,46] isolated from *Paris formosana Hayata* (Liliaceae), which has been used as a folk remedy for snake-bite toxicity and tumors. Various edible plants, such as soybeans and chickpeas, contain saponins [47]. Our previous report demonstrated that formosanin C does not exert adverse effects on non-tumorigenic human cells, including human peripheral blood mononuclear cells and human umbilical vein endothelial cells [25]. Xiao et al. also demonstrated that formosanin C exerts potent inhibitory effects on tumor cell growth without causing cytotoxicity to various normal cell types, including human bronchial cells, human meningeal cells, and vascular smooth muscle cells [48]. In an animal model, the administration of formosanin C (5 mg/kg/every other day via intraperitoneal injection) was well tolerated by C3H/HeN mice, as evidenced by the absence of body weight loss [46]. Despite its non-toxic profile, formosanin C has demonstrated anti-tumor activity. It inhibited the proliferation of ovarian cancer SKOV3 cells [45] and promoted apoptosis in CRC HT-29 cells [25]. Furthermore, in vivo studies using a xenograft mouse model of human ovarian SKOV3 cancer showed that formosanin C significantly reduced both tumor volume and weight [45]. Additionally, formosanin C enhanced the efficacy of 5-fluorouracil in treating hepatoma in MH134-transplanted mice, likely through immune modulation mechanisms that suppressed tumor cell growth [46]. Our previous report indicates that human hepatocellular carcinoma HepG2 cells with an increased expression of nuclear receptor co-activator 4 (NCOA4), a cargo receptor of ferritinophagy, and reduced expression of FTH1, a subunit of iron storage protein ferritin, are more prone to formosanin C-induced ferroptosis than Hep3B cells [29]. Using metastatic hormone receptor-positive (HR^+^) and human epidermal growth factor receptor 2-negative (HER2^−^) breast cancer cells, Pottier et al. revealed that the protein expression of GPX4 was increased in parental cells (e.g., CAMA1, T47D, and ZR75.1) after treatment with a palbociclib and fulvestrant (PF) combination, and in PF-resistant cells (e.g., CAMA1-PFR, T47D-PFR, and ZR75.1-PFR). Silencing GPX4 expression decreased cell proliferation in parental and PF-resistant cells. Moreover, the PF-resistant CAMA1-PFR and T47D-PFR cells, but not the parental CAMA1 and T47D cells, were extremely sensitive to RSL3, a GPX4 inhibitor. These data highlight the role of GPX4 protein, the main protector against ferroptosis, in drug and ferroptosis resistance [49]. In addition, stronger formosanin C-triggered ferroptosis was observed in human triple-negative breast MDA-MB-231 cancer, the most lethal subtype, than in luminal A breast MCF-7 cancer [16]. Altogether, we herein demonstrate formosanin C-activated ferroptosis in CRC cells with p53 and oncogenic KRAS. From the perspective of precision medicine, it is of interest for future interventions to consider formosanin C for counteracting the distinctive molecular aberrations in different cancer types.

## 5. Conclusions

The present study demonstrated that formosanin C may be an effective ferroptosis inducer in CRC cells with p53 or oncogenic KRAS, and our novel gene-expression screening platform via the integrated analysis of differentially expressed genes and pathways may allow for the precise identification of potential naturally occurring compounds capable of targeting specific diseases.

## Data Availability

The data that support the findings of this study are available from the corresponding authors upon request.

## Supplementary Materials

The following supporting information can be downloaded at https://www.mdpi.com/article/10.3390/antiox14081027/s1. Table S1: Gene symbols and their corresponding NCBI gene accession numbers (Section 2.1).

## Abbreviations

Gene: *ACO1*, aconitase 1; *ACSL4*, acylCoA synthetase long chain family member 4; *APC*, adenomatous polyposis coli; *FTH1*, ferritin heavy chain 1; *GCLM*, glutamate–cysteine ligase modifier subunit; *HMOX1*, heme oxygenase 1; *IREB2*, iron responsive element binding protein 2; *NRF2*, nuclear factor erythroid 2-related factor 2; *SLC7A11*, solute carrier family 7 member 11; *SLC40A1*, solute carrier family 40 member 1; *TFRC*, transferrin receptor; *TP53*, tumor protein p53. Methods: ATCC, American Type Culture Collection; CI, combination index; CRC, colorectal cancer; GAPDH, glyceraldehyde-3-phosphate dehydrogenase; GPX4, glutathione peroxidase 4; HER2^−^, human epidermal growth factor receptor 2-negative; HR^+^, hormone receptor-positive; KD, knockdown; LD, lipid droplet; NCOA4, nuclear receptor co-activator 4; OE, overexpression; RIPA, radioimmunoprecipitation assay; SDS-PAGE, sodium dodecyl sulfate–polyacrylamide gel electrophoresis; SEMs, standard errors of the means; Syn, synergism. Chemical compounds: FC, formosanin C; Fer-1, ferrostatin-1; MTT, 3-[4,5-dimethylthiazol-2-yl]-2,5-diphenyltetrazolium bromide; PF, palbociclib and fulvestrant; PPI, polyphyllin I; ROS, reactive oxygen species; RSL3, 1S,3R-Ras-selective lethal small molecule 3. Databases: cbioportal, cBio Cancer Genomics Portal; CGP, chemical and genetic perturbations; CLUE, Connectivity Map and Library of Integrated Network–Based Cellular Signatures Unified Environment; CPDB, ConsensusPathDB; DEGs, differentially expressed genes; DepMap, Dependency Map; GDC–TCGA, Genomic Data Commons–The Cancer Genome Atlas; GSEA, Gene Set Enrichment Analysis; NES, normalized enrichment score; TCGA–COAD, The Cancer Genome Atlas Colon Adenocarcinoma; UCSC, University of California at Santa Cruz.
