# Supplementary material for: Using Integrated Bioinformatics Analysis to Identify Saponin Formosanin C as a Ferroptosis Inducer in Colorectal Cancer with p53 and Oncogenic KRAS
Source: Antioxidants (Basel). 2025 Aug 21;14(8):1027. doi: 10.3390/antiox14081027 (PMC12383135; doi:10.3390/antiox14081027)
Supplement: Supplementary file 1 [file antioxidants-14-01027-s001.zip › antioxidants-3691233-supplementary.pdf]

**Table S1.** Gene symbols and their corresponding NCBI gene accession numbers

| Gene symbol | Gene name | NCBI gene ID |
|---|---|---|
| *TP53* | Tumor protein p53 | 7157 |
| *KRAS* | Kirsten rat sarcoma viral oncogene homolog | 3845 |
| *NRAS* | Neuroblastoma RAS viral (V-Ras) oncogene homolog | 4893 |
| *HRAS* | V-Ha-Ras harvey rat sarcoma viral oncogene homolog | 3265 |
| *BRAF* | v-raf murine sarcoma viral oncogene homolog B1 | 673 |
| *PI3KCA* | Phosphatidylinositol-4,5-bisphosphate 3-kinase | 5290 |
| *TFRC* | Transferrin receptor 1 | 7037 |
| *APC* | Adenomatous polyposis coli | 324 |
| *ACO1* | Aconitase 1 | 48 |
| *IREB2* | Iron responsive element binding protein 2 | 3658 |
| *SLC7A11* | Solute carrier family 7 member 11 | 23657 |
| *FTH1* | Ferritin heavy chain 1 | 2495 |
| *ACSL4* | Acyl-CoA synthetase long chain family member 4 | 2181 |
| *NRF2* | Nuclear factor erythroid 2-related factor 2 | 4780 |
| *GCLM* | Glutamate-cysteine ligase modifier subunit | 2737 |
| *HMOX1* | Heme oxygenase 1 | 3162 |
| *SLC40A1* | Solute carrier family 40 member 1 | 30061 |
